# Novel Materials through Non-Hydrolytic Sol-Gel Processing: Negative Thermal Expansion Oxides and Beyond

**DOI:** 10.3390/ma3042567

**Published:** 2010-04-06

**Authors:** Cora Lind, Stacy D. Gates, Nathalie M. Pedoussaut, Tamam I. Baiz

**Affiliations:** 1 Department of Chemistry, The University of Toledo, Toledo, OH 43606, USA; E-Mail: tamam.baiz@rockets.utoledo.edu (T.I.B.); 2 14841 Hubbell Road, Detroit, MI 48227, USA; E-Mail: stacy.gates@gmail.com (S.D.G.); 3 Canoio, 31530 Bretx, France; E-Mail: npedous@rockets.utoledo.edu (N.M.P.)

**Keywords:** non-hydrolytic sol-gel, metal oxides, negative thermal expansion, metal sulfides

## Abstract

Low temperature methods have been applied to the synthesis of many advanced materials. Non-hydrolytic sol-gel (NHSG) processes offer an elegant route to stable and metastable phases at low temperatures. Excellent atomic level homogeneity gives access to polymorphs that are difficult or impossible to obtain by other methods. The NHSG approach is most commonly applied to the preparation of metal oxides, but can be easily extended to metal sulfides. Exploration of experimental variables allows control over product stoichiometry and crystal structure. This paper reviews the application of NHSG chemistry to the synthesis of negative thermal expansion oxides and selected metal sulfides.

## 1. Introduction 

Advanced materials play an important role in everyday life of modern society. Solid-state inorganic materials transcend all aspects of our lives, from structural materials to specialized compounds used in electronics, optics, and energy applications. The oldest methods of obtaining inorganic solids are mining of naturally occurring compounds, and traditional ceramic synthesis. This limits accessible phases to thermodynamically stable polymorphs under given temperature-pressure conditions.

The advent of more sophisticated applications over the past half century has led to tremendous interest in alternative synthetic routes. Careful modification of stoichiometry and doping, formation of desired particle size and morphology, and control of crystal structure are crucial targets in many cases. A variety of soft chemistry or “chimie douce” routes have been developed, which give access to stable and metastable polymorphs [1,2,3,4,5,6,7,8,9,10,11,12]. Methods include chemical vapor deposition [13], ion exchange [14], intercalation [15], flux synthesis [12,16], topotactic transformations of precursors [12,17], hydrothermal synthesis [12,18,19], and sol-gel reactions [8,12,20,21]. A common feature of these methods is the use of significantly lower reaction temperatures. This can be achieved through reactions in the gas or liquid phase, which provide much faster diffusion compared to traditional solid-state approaches. Other reactions start from precursors that already exhibit the desired atomic level connectivity of the target material, and in some cases contain important structural features similar to the product polymorph of interest [12,17,22].

**Figure 1:**
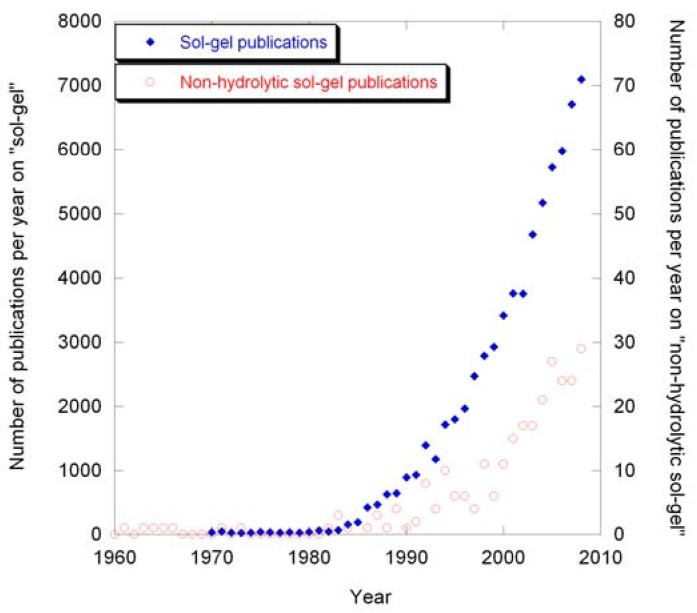
Number of publications per year on “sol-gel” and “non-hydrolytic sol-gel”.

Sol-gel routes are attractive, as they provide a solution based approach to many simple and complex materials [8,12,20,21]. The reaction outcome can often be influenced by careful control of several reaction variables. Another advantage of sol-gel methods is the wide range of accessible shapes, which include fine powders, fibers, thin films, xerogels, aerogels, and monoliths [8]. Sol-gel processes can be further divided into traditional or aqueous sol-gel routes [8,21,23], and non-hydrolytic sol-gel reactions [24,25,26]. Traditional sol-gel routes rely on hydrolysis and condensation reactions between metal alkoxides and small amounts of water in organic solvents. In this process, the breakage of a metal-oxygen bond is the rate limiting step, resulting in significantly different kinetics for different metals. The condensation results in formation of a sol, which transforms into a gel as the polymerization proceeds. This method was already known in the second half of the 1800’s, but only became widely explored in the mid 1980’s, as evidenced by the exponential increase in the number of publications per year over the past 25 years (Figure 1). Conventional sol-gel routes have been applied to commercially important binary oxides like silica, titania, and zirconia [27,28], as well as to ferroelectrics [29], superconductors [12], and other classes of materials.

**Figure 2:**
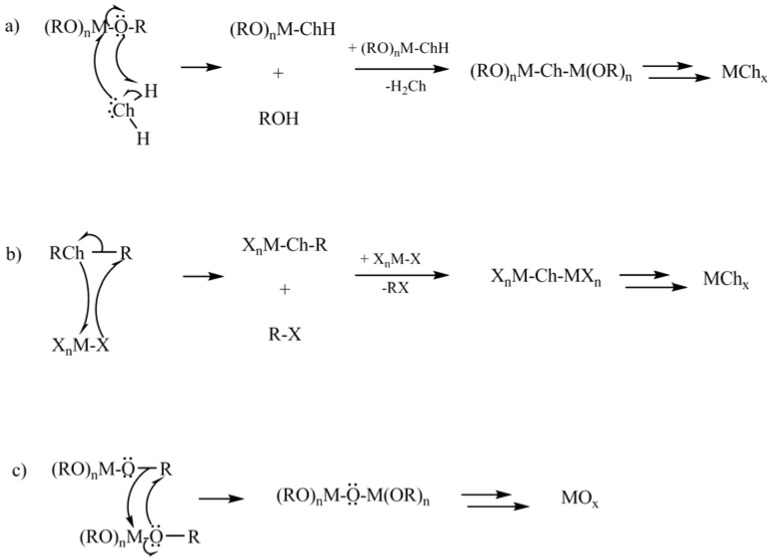
Scheme of (a) conventional and (b) and (c) non-hydrolytic sol-gel reactions. The reaction paths (b) and (c) show the mechanisms of NHSG reactions starting from metal halides, and the special case of the Bradley ether elimination reaction. Ch = chalcogenide (O, S), X = halide.

A limitation of aqueous sol-gel processes arises for mixed metal oxides where individual components show very different hydrolysis rates (e.g., ZrTiO_4_) [30]. In such cases, complexing agents are required to slow down the hydrolysis of the more reactive precursor. This can make starting materials expensive for some systems, and often results in poor control of homogeneity. An elegant alternative is the use of a non-hydrolytic sol-gel (NHSG) route, in which metal halides are reacted with secondary or tertiary ethers as the oxygen source [24,31,32,33,34,35]. Alternatively, reactions can use metal alkoxide starting materials directly, and proceed via ether elimination, as is the case in the Bradley reaction [36]. The Bradley reaction can present a competing reaction path during the reaction of metal halides with ethers, as metal alkoxides with secondary or tertiary alkyl groups are formed *in situ* during NHSG processes [37]. Compared to conventional sol-gel processes, NHSG reactions follow a different mechanism, leading to significantly less dependence on the identity of the metal ions (Figure 2). The mechanism of this process was established through NMR experiments, and involves the *in situ* formation of metal alkoxides, followed by nucleophilic attack of a halide on the alkyl groups [38]. The rate limiting reaction step is the breaking of a carbon-oxygen bond, which results in comparable reaction rates for most soluble metal halides. An EXAFS study by Xu *et al*. showed that amorphous zirconium titanate gels prepared by NHSG chemistry contained well-defined Zr-O-Ti linkages, in contrast to gels obtained by conventional sol-gel processes [39].

The first reports of non-hydrolytic sol-gel syntheses date back to the 1950’s [40], when the preparation of silica and boron phosphate was reported. The method received very little attention until the 1990’s, when French researchers explored the applicability of the process to a number of binary and ternary oxide systems, and coined the name “non-hydrolytic sol-gel chemistry” [24,31,32,33,34,38,41,42]. While the number of publications on NHSG routes is about two orders of magnitude lower than for conventional sol-gel processes, a significant increase in the number of publications per year has been observed over the past two decades. Previously unaccessible compositions and polymorphs have been discovered through this method.

The preparation of non-oxide materials is underexplored compared to oxides, owing to the in many cases more facile synthesis and handling of the latter. This is especially true for the area of low temperature reactions. However, there are emerging fields that require or can benefit from non-oxide ceramics [43]. Examples include solar cells [44], catalysis [45,46,47], extremely hard materials [48,49,50], and optoelectronic applications [51]. Sol-gel related methods have been successfully extended to the synthesis of selected carbides, nitrides, and sulfides. Sulfides have received the most attention among these materials because of their catalytic [45,46,47,52,53,54], electronic [55,56] and optical [57,58] properties. Metal sulfides are used as catalysts for hydrodesulfurization in the petroleum industry, as lubricants, and as electrodes in lithium batteries [59,60,61]. Other potential applications include optical materials in IR windows, solar cells, lasers, phosphors, and light emitting diodes [57,58,62]. 

Sol-gel methods to metal sulfides can be divided into thio-sol-gel routes, which are the equivalent of conventional sol-gel reactions to metal oxides, and a process similar to NHSG approaches to metal oxides (Figure 2). In thio-sol-gel reactions, metal alkoxides are reacted with controlled amounts of hydrogen sulfide. Incorporation of oxygen from the alkoxide groups is often observed in the products, and heat treatment in H_2_S is necessary to obtain oxygen-free materials. The heating time and temperature vary depending on the metals involved, and sulfides of oxophilic metals frequently cannot be obtained without oxide impurities [63]. In the late 1980’s, Schleich *et al*. investigated the reaction of metal halides with organic sulfur sources [64,65,66,67,68]. This procedure is comparable to NHSG routes developed for metal oxides [24,25,69], and will be referred to as “non-hydrolytic” sol-gel approach to metal sulfides. 

## 2. Non-Hydrolytic Sol-Gel Synthesis of Metal Oxides

Non-hydrolytic sol-gel chemistry offers an elegant alternative to conventional sol-gel routes in multicomponent systems with considerably different reaction rates of individual precursors. The name non-hydrolytic sol-gel chemistry for reactions of metal halides with ethers in organic solvents was suggested by a group of French scientists around André Vioux, who reported investigations on the synthesis of SiO_2_ [70], Al_2_O_3_ [38], TiO_2_ [42], Nb_2_O_5_, MoO_3_, ZrO_2_, WO_3_, Fe_2_O_3_, V_2_O_5_ [32], Al_2_TiO_5_ [71,72] and ZrTiO_4_ [33] in the 1990’s. The term NHSG reaction has not only been applied to systems that result in monolithic gels, but based on a common reaction mechanism also to precursors that yield precipitates. This method was later adapted to other complex oxide systems [73,74,75,76], as well as the preparation of hybrid organic-inorganic gels [77,78,79,80]. Our group has been exploring NHSG routes to negative thermal expansion (NTE) materials in the A_2_M_3_O_12_ family (A = trivalent metal; M = Mo, W) [81,82,83].

In a typical NHSG synthesis, metal halide starting materials and a dry, inert solvent are added to a glass ampoule inside a glovebox. The ampoule is capped with a septum and attached to a Schlenk line. Isopropyl ether is added by syringe. The ampoule is evacuated, sealed and heated to 100–150 °C for one week. Amorphous powders or tars are recovered by filtration or evaporation, respectively. Reaction variables include initial metal ratios, solvent choice, and heating temperature of the ampoule.

### 2.1. NHSG Routes to A_2_M_3_O_12_ Materials

The A_2_M_3_O_12_ family of compounds adopts a number of different structure types due to the large range of ionic radii found for trivalent metals (0.535 Å for Al^3+^ to 1.061 Å for La^3+^). For most structures, the M^6+^ cation is tetrahedrally coordinated. On the A-site, different size cations result in different coordination environments, with the larger A^3+^ cations generally adopting high coordination numbers (7 or 8), while smaller cations usually prefer 6-coordinate structures [84]. Trivalent d- and p-block elements are commonly found in octahedral coordination, forming corner-sharing frameworks with monoclinic (space group P 2_1_/a) or orthorhombic (space group Pbcn) structures. These two polymorphs share a close structural relationship, and many compositions undergo a phase transition from the monoclinic to the orthorhombic phase upon heating [85,86]. Negative thermal expansion has only been observed in the orthorhombic Pbcn polymorph, making it the target structure for the synthesis of NTE materials [87,88,89]. The exact expansion and phase transition behavior is strongly dependent on the identity of the cations incorporated in the structure [89].

**Table 1:** Optimized reaction conditions for the non-hydrolytic sol-gel synthesis of A_2_M_3_O_12_ and AA’M_3_O_12_ compositions (A = trivalent metal or Mg; A’ = Zr, Hf; M = Mo, W).

Target Compound	A(:A’):M Ratio (mmol)	Solvent (amount/mL)	Reaction T (°C)	Crystallization T (°C)
Al_2_Mo_3_O_12_	2:3	CHCl_3_ (10)	130	700
Sc_2_Mo_3_O_12_	2:3	CHCl_3_ (10)	130	700
Fe_2_Mo_3_O_12_	1:1.5	CH_3_CN (10)	130	500
Ga_2_Mo_3_O_12_	2:2	CH_3_CN (10)	110	600
In_2_Mo_3_O_12_	2:3	CH_3_CN (5)	130	600
Y_2_Mo_3_O_12_	2:3	CH_3_CN (5)	110	450* ( Pbcn); 500 (Pba2)
MgZrMo_3_O_12_	1:1:2.5	CHCl_3_;THF (8; 3)	130	600
MgHfMo_3_O_12_	1:1:2.5	CHCl_3_;THF (8; 3)	130	600
MgZrW_3_O_12_	1:1:2.5	CHCl_3_;THF (8; 3)	130	600
MgHfW_3_O_12_	1:1:2.5	CHCl_3_;THF (8; 3)	130	600

* Kinetic crystallization during *in situ* heat treatment. This phase is thermodynamically stable above 550 °C.

A number of A_2_M_3_O_12_ compositions are thermodynamically stable and accessible by high temperature routes. However, some cations can not be incorporated into the framework unless low temperature approaches are used. In addition, fine tuning of properties like phase transition behavior and magnitude of expansion coefficients may be possible for systems with mixed cation occupancy on the A and M sites. In such systems, good homogeneity is important. Solution based routes are more likely to produce homogeneous mixed cation systems than traditional ceramic approaches.

NHSG synthesis of A_2_M_3_O_12_ compositions can be carried out by reacting metal chlorides and diisopropyl ether in sealed glass ampoules at elevated temperatures. While initial reports in the 1990’s mainly used chloroform, dichloromethane or carbon disulfide as solvents, we have found that acetonitrile gives better solubility for several starting halides. The raw, as recovered materials are generally amorphous and contain significant amounts of residual organics, which can be removed by heat treatment. Crystallization of oxide phases is observed at temperatures ranging from 400 to 700 °C. Optimized reaction conditions have been established for a number of previously reported A_2_M_3_O_12_ polymorphs (Table 1). In addition, several new compositions and polymorphs were discovered using NHSG chemistry, which will be discussed in more detail. 

### 2.2. Gallium Molybdate Ga_2_Mo_3_O_12_

Prior to our report on the synthesis and characterization of Ga_2_Mo_3_O_12_ in 2006 [83], no ternary gallium molybdenum oxide was known. The mixed metal oxide is thermodynamically disfavored compared to a mixture of the binary components, ruling out high temperature synthetic approaches. Attempts to prepare a ternary compound by coprecipitation or hydrothermal methods also resulted in phase separation and crystallization of binary oxide mixtures.

Non-hydrolytic sol-gel reactions of a 2:3 mixture of GaCl_3_ and MoCl_5_ in CHCl_3_ or CH_3_CN resulted in dark, amorphous powders or tars, which crystallized into a mixture of MoO_3_ and monoclinic Ga_2_Mo_3_O_12_ upon heating. Elemental analysis confirmed that the powder contained an excess of molybdenum, suggesting that the gallium precursor may be more soluble or react incompletely. Acetonitrile is the preferred solvent for the synthesis of Ga_2_Mo_3_O_12_, as GaCl_3_ appears to undergo a side reaction in chloroform that produces hydrogen gas. The procedure was optimized to obtain phase pure Ga_2_Mo_3_O_12_, which was achieved by heating a sealed ampoule containing 2 mmol of GaCl_3_ and MoCl_5_ each in 10 mL of acetonitrile at 110 °C for 1 week. Samples contained about 40 weight% residual organics, which were lost by heating to ~500 °C. Crystallization of Ga_2_Mo_3_O_12_ was observed between 500 and 600 °C. Rietveld refinement confirmed that this phase is isostructural with monoclinic Al_2_Mo_3_O_12_. No phase changes were observed upon heating to 650 °C, at which temperature the material decomposed into Ga_2_O_3_ and MoO_3_. Monoclinic Ga_2_Mo_3_O_12_ exhibits positive expansion, and undergoes two reversible pressure-induced phase transitions at 3.2 and 4.1 GPa [83].

### 2.3. Low-Temperature Phase of Yttrium Molybdate Y_2_Mo_3_O_12_

Yttrium molybdate, Y_­2_Mo_3_O_12_, has been known for a long time [84,90]. The compound can be synthesized from binary oxides at high temperatures. It was originally described as adopting a tetragonal structure with 7-coordinate yttrium at room temperature, and to transform to an orthorhombic Pbcn phase with 6-coordinate yttrium at higher temperatures [84]. More recent literature exclusively assigned Y_2_Mo_3_O_12_ to the Pbcn structure at all temperatures, and reported NTE behavior over a wide temperature range. At temperatures below 130 °C, absorption of atmospheric moisture led to formation of a trihydrate, Y_2_Mo_3_O_12_·3H_2_O [91]. 

NHSG processing of Y_­2_Mo_3_O_12_ resulted in crystallization of two different orthorhombic polymorphs, which adopt structures with 6-coordinate yttrium in space group Pbcn, and 7-coordinate yttrium in space group Pba2, respectively [82]. The Pba2 phase is isostructural to orthorhombic Tb_2_Mo_3_O_12_ and Gd_2_Mo_3_O_12_ [92]. Coexistence of the two phases was observed in samples that were heated to 500–600 °C and slow cooled. Heat treatments to temperatures of 700 °C or higher resulted in single phase Pbcn samples. *In situ* X-ray studies revealed that crystallization of the Pbcn polymorph from the amorphous raw material started at 450 °C. A variable temperature diffraction study of a mixed phase sample, however, showed transformation of the Pba2 phase into the Pbcn structure at 550 °C. These results suggest that formation of the Pbcn polymorph is kinetically favored, allowing crystallization in a temperature range where it is thermodynamically metastable with respect to the Pba2 phase. The Pba2 and Pbcn structures are thermodynamically stable below and above 550 °C, respectively. 

**Figure 3:**
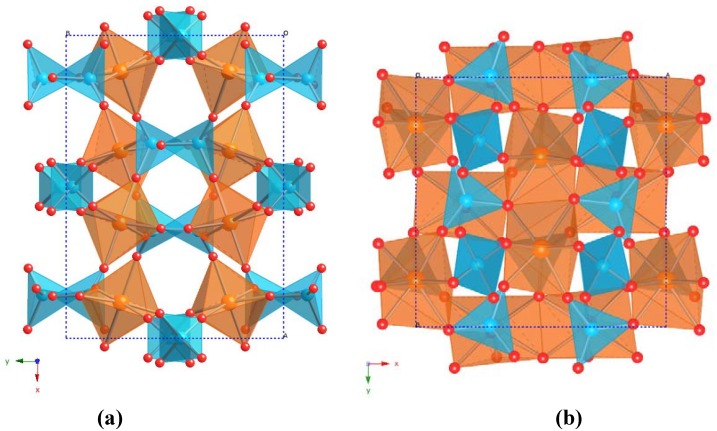
Crystal structures of the (a) Pbcn and (b) Pba2 polymorphs of Y_2_Mo_3_O_12_ viewed down the z axis. Blue tetrahedra are MoO_4_ units, and orange polyhedra represent 6- and 7-coordinate yttrium, respectively.

**Figure 4:**
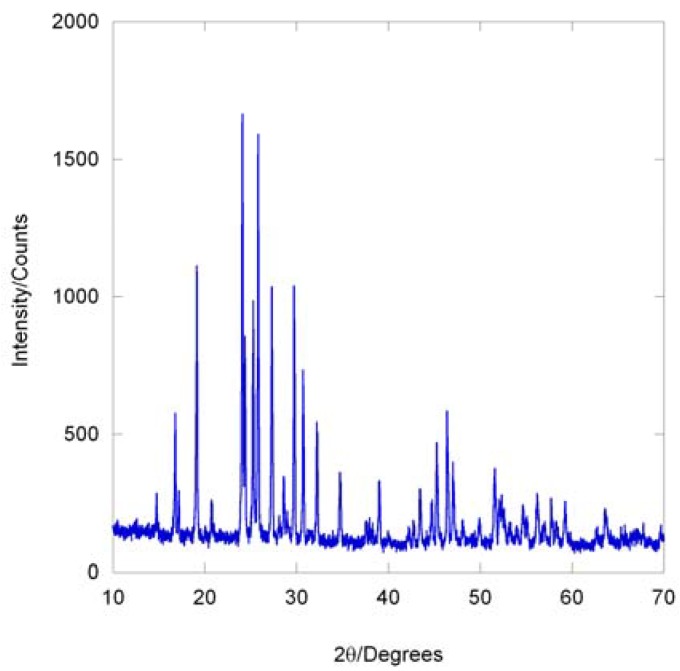
X-ray diffraction pattern of single phase Pba2-Y_2_Mo_3_O_12_ prepared by annealing at 530 °C for 2 months.

Interconversion of the two orthorhombic Y_2_Mo_3_O_12_ polymorphs requires significant changes in local bonding, as the coordination number of yttrium changes from 7 to 6 between the Pba2 and Pbcn structures. The denser Pba2 polymorph contains edge shared polyhedra, while the Pbcn phase is composed of a corner sharing network of polyhedra (Figure 3). Phase pure Pbcn-Y_2_Mo_3_O_12_ is easily obtained by heating to high temperatures, whereas formation of single phase Pba2-Y_2_Mo_3_O_12_ requires several months of annealing at 530 °C (Figure 4). The crystallization of both polymorphs at temperatures below 500 °C is an excellent demonstration of the atomic level homogeneity of samples prepared by NHSG chemistry.

### 2.4. MgAM_3_O_12_ Compositions (A = Zr, Hf; M = Mo, W)

Most research in the A_2_M_3_O_12_ family has focused on compounds containing single A-site and M-site cations. Materials with mixed occupancy of two different trivalent cations on the A-site have also been reported [87,89]. Compositions with differently charged cations on the A-site were not known until 2004, when Suzuki *et al.* reported the synthesis of MgHfW_3_O_12_ through extensive ball milling and heat treatment [93]. Detailed structural studies by our group showed that MgHfW_3_O_12_ and MgZrW_3_O_12_ crystallize in a monoclinic phase isostructural to many A_2_M_3_O_12_ compositions with trivalent cations below 125 °C [94]. Neutron diffraction studies revealed that the +2 and +4 cations adopt an ordered arrangement. At higher temperatures, the compounds undergo a phase transition to an orthorhombic phase. While the lattice constants resemble those of other orthorhombic A_2_M_3_O_12_ materials, the symmetry is lowered to Pnma or Pna2_1_. The atomic level structure of this new polymorph, which exhibits NTE, has not been determined to date.

**Figure 5:**
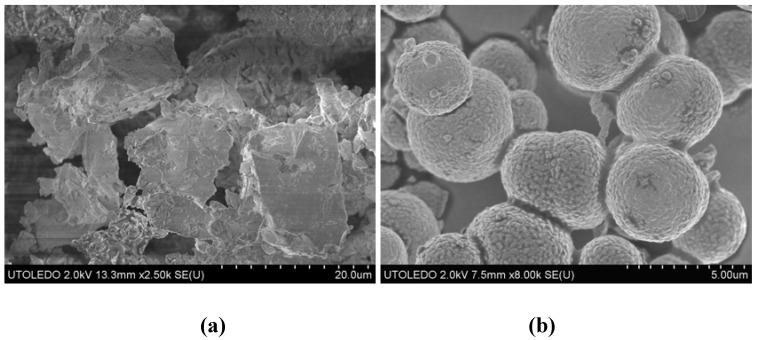
SEM images of MgZrM_3_O_12_ prepared by (a) ball milling and high temperature treatment and (b) non-hydrolytic sol-gel chemistry.

The high temperature synthesis (24 h at 1050–1100 °C) of single phase MgAW_3_O_12_ (A = Zr, Hf) is challenging. Extended ball milling is necessary to obtain the quarternary oxide at all, and the sintering temperature has to be very tightly controlled to avoid formation of other binary or ternary oxide phases. To facilitate preparation of MgAW_3_O_12_ compositions, and to extend the studies to the corresponding molybdates, we decided to explore NHSG processing of the target compounds [81]. As ZrCl_4_ and HfCl_4_ show very limited solubility in CHCl_3_, A(O*i*Pr)_4_·HO*i*Pr were chosen as starting materials and dissolved in redistilled THF. MgCl_2_ and MoCl_5_ or WCl_6_, respectively, were suspended in CHCl_3_ (see Table 1). Reactions of stoichiometric 1:1:3 mmol ratios of MgCl_2_, A(O*i*Pr)_4_·HO*i*Pr and MoCl_5_ or WCl_6_ resulted in cocrystallization of MgAM_3_O_12_ and MO_3_ (M = Mo, W) when the amorphous raw powders were heat treated to 600 °C. As in the case of GaMo_3_O_12_, this could be a result of different solubilities and reactivities of the starting metal compounds. Phase pure MgAM_3_O_12_ samples could be obtained by reducing the amount of Mo/W starting material to 2.5 mmol. Excellent crystallinity could be achieved for the tungstates by 30 min heat treatment to 1050 °C. The corresponding molybdates could also be obtained, but decomposed at ~800 °C. All samples were composed of micron to submicron size particles instead of the large, sintered pieces observed during high temperature syntheses (Figure 5). The raw precipitates contained spherical particles up to 5 μm in size. The external particle shape was preserved during heating, but a large number of nuclei formed in each particle, resulting in spherical agglomerates of submicron crystallites.

## 3. Non-Hydrolytic Sol-Gel Synthesis of Metal Sulfides

Non-oxide materials have increasingly attracted the interest of researchers, as many advanced applications in optoelectronics, catalysis, energy and other fields require properties that are difficult or impossible to achieve with oxides [43]. Metal sulfides have been explored for their catalytic, optical and electronic properties. While these materials can be air-sensitive, the improvement in performance offsets the difficulties in handling and protecting the compounds. Many applications depend on the quality of the sulfide materials. Homogeneity, low oxidation state, glassy structure, or high surface area are often desirable. Low temperature routes like chemical vapor deposition [95,96,97,98,99], precipitation [100,101,102], and sol-gel methods [103,104,105,106] have been explored to overcome diffusion-related kinetic barriers, and to access metastable phases or tailor properties like grain size and surface area. 

“Non-hydrolytic” sol-gel approaches provide a solution based route to metal sulfides that rigorously excludes oxygen during all stages of the process. This process was first explored by Schleich *et al.* [64,65,66,67,68], who investigated the reaction of several transition metal halides with different organic sulfur sources in CH_2_Cl_2_. Sulfurizing agents included hexamethyldisilthiane, mercaptanes, di-*tert*-butyl disulfide and di-*tert*-butylsulfide (DTBS). Reactions were carried out between room and reflux temperature. The recovered products were generally amorphous, and could be crystallized through heating. Thermodynamically stable sulfide phases were obtained after heat treatments to 500 to 1000 °C (TiS_2_, Ti_3_S_4_, V_5_S_8_, NbS_2_, Cr_2_S_3_, MoS_2_, WS_2_, FeS) [64,66,67]. Most of this early work was focused on the characterization of the amorphous powders through NMR, IR and elemental analysis. Based on Schleich’s original results, we have started thorough explorations of the available variable space, especially with respect to crystallization behavior of the recovered powders at low temperatures. Our initial results demonstrate that NHSG reactions to metal sulfides have tremendous potential for the synthesis of unusual or previously unknown polymorphs.

For a typical NHSG synthesis of a transition metal sulfide, a few millimole of a metal halide starting material are mixed with dry chloroform or acetonitrile inside a glovebox. DTBS is added in stoichiometric amounts or in excess. The ampoule is sealed and heated for a week. Products are recovered by filtration inside the glove box. The powders are protected from atmosphere for all subsequent heating and characterization steps. Heat treatments are carried out under argon flow in a tube furnace. The tube is equipped with valves and can be packed inside the glovebox. Air sensitive sample holders are available for X-ray characterization in inert atmosphere.

### 3.1. Synthesis of Troilite with Unusual Stoichiometry

Iron readily forms a number of sulfides and disulfides [107,108,109]. Many of the sulfides have similar stoichiometries, close to FeS. A thiospinel, greigite, Fe_3_S_4_, is also known, which contains defined quantities of Fe^2+^ and Fe^3+^. The description of the Fe-S phase diagram is complicated by the fact that in addition to a large number of mineral phases with similar composition, individual mineral phases can also exhibit a plethora of distinct phases depending on exact composition and temperature. Among these phases, the structures of hexagonal and monoclinic pyrrhotites are based on the nickel arsenide structure, which consists of a hexagonal close packed array of Ni atoms with As atoms occupying the octahedral holes. Pyrrhotites adopt a large number of Fe_1-x_S compositions, many of which form related but distinct structures due to an ordered arrangement of iron vacancies [110,111]. The pyrrhotite phase diagram is very complex, with superstructures described by their relationship to the unit cell of the parent NiAs structure. The stoichiometric or near stoichiometric (generally less than 5% iron vacancies) end member, troilite, does not form naturally on Earth, but is found on the moon, in planetary rock, and in meteorites [112,113]. It serves as a geochemical marker, and can be used to reconstruct the temperature/pressure history of planets. Synthetic routes to troilite require either extended heating at high temperatures, or long periods of high energy ball milling [114,115].

NHSG reactions were carried out with FeCl_3_ and FeCl_2_. No iron sulfides could be recovered for FeCl_3_ under any conditions investigated. FeCl_2_ resulted in an explosion when heated in CHCl_3_, but dissolved and reacted readily with DTBS in CH_3_CN when temperatures between 110 and 150 °C were used. No powders could be recovered at lower temperatures due to formation of soluble complexes with acetonitrile. Metal to sulfur ratios were varied from 1:1 to 1:7, and metal concentrations between 0.2 M and 0.3 M were chosen.

**Figure 6:**
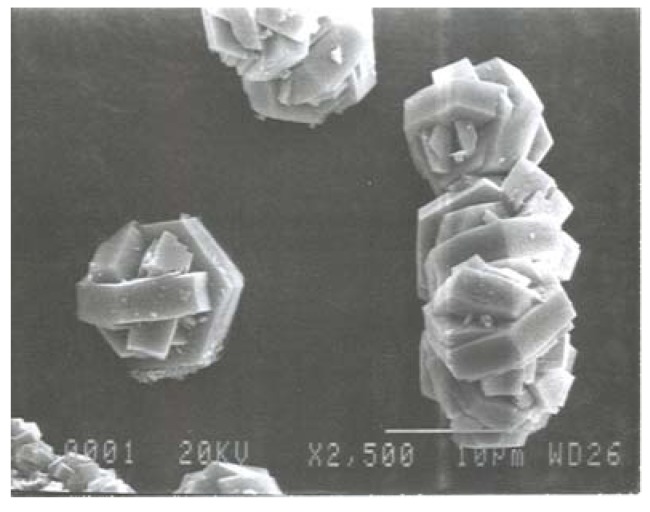
Electron micrograph of intergrown hexagonal troilite platelets.

All as-recovered powders were crystalline, and contained less than 1.5 wt% carbon. X-ray patterns revealed the presence of two iron sulfide phases, greigite (Fe_3_S_4_) and troilite (Fe_1-x_S), in varying ratios. It was found that lower reaction temperatures favored the formation of troilite, while a reaction carried out at 150 °C produced greigite as the major phase. Heat treatments of the as-recovered powders resulted in sulfur loss and conversion of the greigite phase to Fe_1-x_S. Energy dispersive X-ray spectroscopy confirmed the presence of two distinct phases in the as-recovered samples with compositions of Fe_3_S_4_ and Fe­_1-x_S, with x-values between 0.09 and 0.16. This was surprising, as troilite usually only forms for compositions with less than 5% iron vacancies [107]. The stoichiometry of Fe_1-x_S can also be calculated from specific peak positions in the X-ray pattern, which gave comparable values between x = 0.11 to 0.13. Rietveld refinements using the troilite structure as a starting model gave excellent fits, and a final refined stoichiometry of Fe_0.89_S to Fe_0.9_S for several samples. Variable temperature diffraction experiments revealed that the troilite phase transformed to monoclinic pyrrhotite of composition Fe_7_S_8_ above 250 °C, as evidenced by peak shape analysis of specific reflections. This confirmed that the samples indeed consisted of troilite with an unusual stoichometry, and that NHSG chemistry provides facile access to small, well-defined hexagonal particles of this phase (Figure 6) [116].

### 3.2. High Pressure Phase of Copper Sulfide

The Cu-S phase diagram is very complex [117]. Copper sulfides form several line compounds, ranging in composition from CuS to Cu_2_S. The richest portion of the phase diagram is based between compositions of Cu_2_S and Cu_1.75_S. Several different compositions, some of which are line compounds, some of which exhibit narrow solid-solutions ranges, and several different polymorphs of identical composition are known. Metastable phases have also been reported, like a tetragonal Cu_1.96_S polymorph formed under pressure (0.1 to 0.25 GPa at temperatures between 100 and 150 °C), which can be quenched to ambient conditions [118].

Experiments in the Cu-S system used two metal precursors, CuCl and CuCl_2_, which were reacted with DTBS in two different solvents, CHCl_3_ and CH_3_CN. Metal chloride starting concentrations varied from 0.2 to 1.0 M, metal to sulfur ratios were chosen between 1:1 and 1:10, and reactions were carried out at 70 to 150 °C.

**Figure 7:**
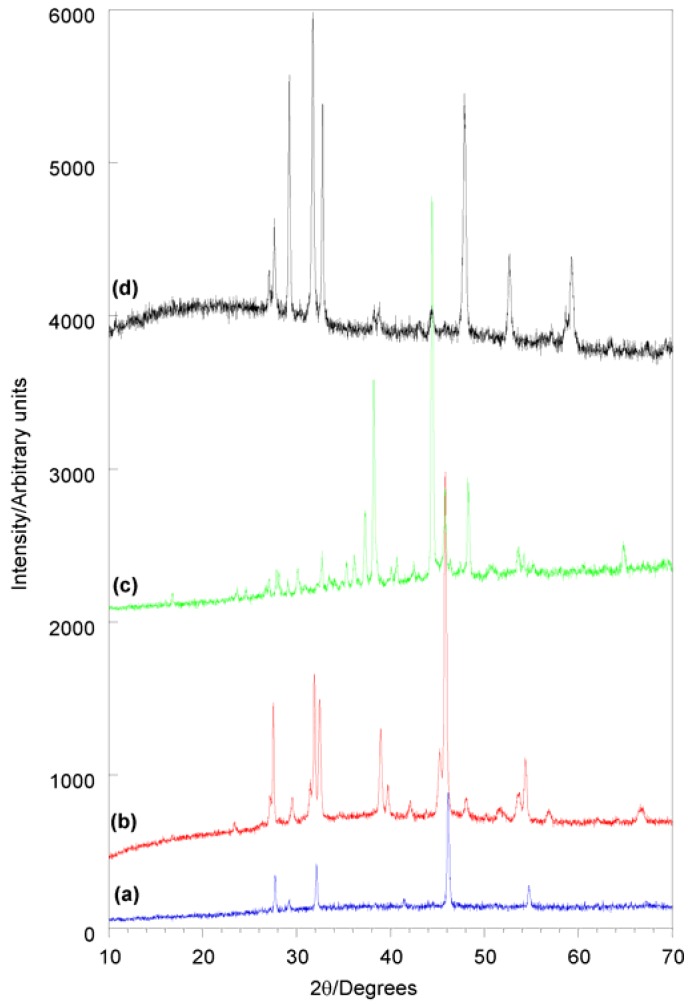
X-ray diffraction patterns of (a) a digenite sample prepared in CHCl_3_ with CuCl, (b) a mixture of digenite and tetragonal chalcocite prepared by heating the sample in (a) to 400 °C for 5 h, (c) a monoclinic chalcocite sample prepared by heating the sample in (a) to 400 °C for 34 h, and (d) a covellite sample prepared with CuCl_2_ in CHCl_3_ at 150 °C.

Similar to the Fe-S system, as-recovered samples were crystalline, although the presence of an amorphous component was evident in many patterns. The crystalline polymorph recovered could be influenced through careful choice of reaction conditions (Figure 7). Crystalline copper sulfide phases were observed for reaction temperatures of 100 °C or higher, while copper chloride starting materials were recovered at 70 °C, indicating that there is a certain activation barrier for the reaction between copper chlorides and DTBS. All reactions starting from CuCl resulted in rhombohedral digenite (Cu_9_S_5_) [119] regardless of solvent and temperature chosen. Reactions carried out with CuCl_2_ in acetonitrile also produced digenite, while those in chloroform yielded mixtures of hexagonal covellite (CuS) [120] and digenite. The relative amounts of the two phases could be influenced by careful choice of reaction temperature, giving pure digenite at 100 °C, and pure covellite at 150 °C. Heating in a tube furnace under flowing argon resulted in transformation of the digenite and covellite phases to chalcocite (Cu_2-x_S) [121,122] at around 400 °C for all samples. This was somewhat surprising, as covellite is stable up to 500 °C, and the digenite structure can be observed between room temperature and 1130 °C. In addition, all but one sample transformed to a phase mixture of the monoclinic ambient pressure and the tetragonal high pressure polymorphs of chalcocite. This high pressure polymorph was the major phase in all heat treated samples, and converted to monoclinic chalcocite upon extended heating at 400 °C. The formation of chalcocite indicates that NHSG prepared copper sulfide samples easily lose sulfur, resulting in sulfides that mainly contain Cu(I). The pure covellite sample transformed to single phase tetragonal Cu_1.96_S. 

The formation of the tetragonal high pressure polymorph in an open heat treatment system (argon flow-through tube furnace) is intriguing, as its preparation usually requires application of pressure to monoclinic chalcocite samples, or direct preparation from the elements in sealed glass tubes under controlled sulfur pressure. This suggests that the atomic ordering favoring the high pressure polymorph may be formed during the initial NHSG reaction in the sealed glass ampoule. The organic solvents used in the NHSG processes generate several bars of autogenous pressure during reactions in sealed glass ampoules at 100–150 °C. While much lower than the pressure required for formation of tetragonal Cu_1.96_S, the structure of the reaction product can be affected by this pressure and favor denser atomic packing within the NHSG product. Such behavior has been observed during the preparation of Y_2_Mo_3_O_12_ by NHSG chemistry in our group, where a denser polymorph (space group Pba2) than the previously reported Pbcn-Y_2_Mo_3_O_12_ was obtained (see 2.3.) [82]. 

### 3.3. New Phase in the Tungsten Sulfide System

The W-S phase diagram contains two binary compounds, congruently melting WS_2_, and WS_3_ with an upper limit of stability around 300–400 °C [123]. WS_3_ is only known as an amorphous material, while crystalline WS_2_ has found widespread industrial use as a dry lubricant and in catalysis. It forms two polymorphs belonging to the rhombohedral and hexagonal crystal systems, which have very similar layered structures. The rhombohedral polymorph is favored at high pressures [124,125].

NHSG reactions in the W-S system were carried out using WCl_6_ and DTBS as metal and sulfur sources, respectively. Neat reactions, and reactions in chloroform and acetonitrile, were carried out. Reaction temperatures ranged from room temperature to 130 °C. The tungsten to sulfur ratio was varied between 1:1 and 1:40. Metal concentrations ranged from 0.02 to 0.6 M.

**Figure 8:**
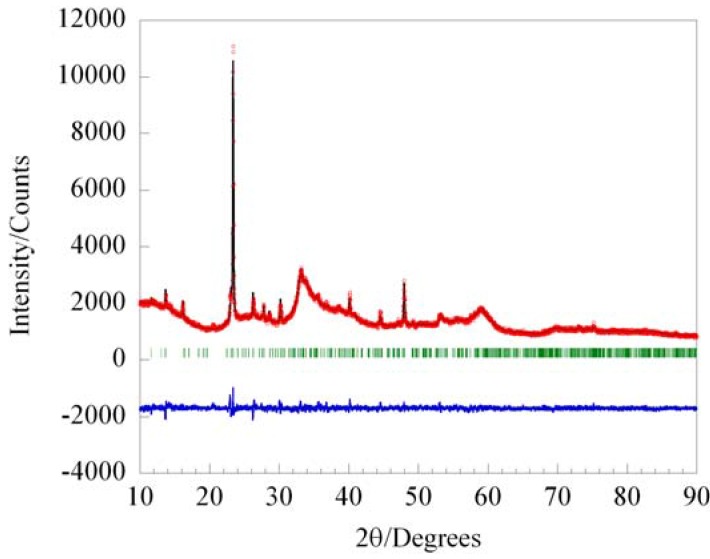
Le Bail fit the new WS_x_ polymorph assuming a monoclinic cell with a = 13.977 Å, b = 3.076 Å, c = 11.104 Å and β = 103.8°.

All as-recovered precipitates were amorphous, with a small amount of elemental tungsten present, indicating partial reduction of the metal halide by DTBS. Most samples required heat treatment to 800 °C to induce crystallization of the thermodynamically stable hexagonal WS_2_ polymorph. For a narrow set of reaction conditions, namely at a metal concentration of 0.2 M in CHCl_3_, sulfur to tungsten ratios of 2 to 4, and using a sealed ampoule at 70 °C, an unknown phase was observed in the X-ray pattern after heating at 500–600 °C. This new polymorph could not be produced as highly crystalline material, all X-ray patterns showed a significant amorphous component. Extended heat treatment at 600 °C or heating to higher temperatures resulted in formation of hexagonal WS_2_. The powder pattern of the new phase could be indexed on a monoclinic unit cell with lattice constants of a = 13.977 Å, b = 3.076 Å, c = 11.104 Å and β = 103.8°. A Le Bail fit using this cell is displayed in Figure 8. CHN analysis on the heat treated powder indicated that the samples contained ~5 wt% residual carbon. It is currently unclear whether this carbon content is part of the crystalline or amorphous phase. Thermogravimmetry and energy dispersive X-ray spectroscopy suggest a W:S ratio of 1:2, which implies a new polymorph of WS_2_. Further refinement of reaction variables may increase the amount of the new crystalline phase compared to the amorphous component, and may ultimately permit determination of its structure.

## 4. Conclusions

Non-hydrolytic sol-gel processes offer a powerful synthetic approach to metal oxides and metal sulfides. Product stoichiometry, and in some cases crystalline structure, can be influenced by careful control of reaction variables. Crystalline materials with small, homogeneous particle size can be obtained reproducibly. The discovery of several new mixed metal oxides (Ga_2_Mo_3_O_12_, Pba2-Y_2_Mo_3_O_12_, MgZrMo_3_O_12_ and MgHfMo_3_O_12_) clearly demonstrates the potential of NHSG methods for the preparation of complex metal oxides. We have also successfully started to adapt NHSG routes to the synthesis of transition metal sulfides. Instead of targeting specific compositions or structures, these experiments were of exploratory nature, with the goal of more fully understanding the accessible phase space. In all systems investigated, fine tuning of experimental variables resulted in formation of unusual known phases (iron deficient troilite Fe_0.9_S, tetragonal chalcocite Cu_1.96_S) or new polymorphs (monoclinic WS_2_). It is likely that many more new phases will be discovered using NHSG chemistry as the potential of these methods for the preparation of metal oxides and sulfides is further explored.
